# Evaluation of a Quadrivalent *Shigella flexneri* Serotype 2a, 3a, 6, and *Shigella sonnei* O-Specific Polysaccharide and IpaB MAPS Vaccine

**DOI:** 10.3390/vaccines12101091

**Published:** 2024-09-24

**Authors:** Emily M. Boerth, Joyce Gong, Becky Roffler, Zoe Hancock, Lydia Berger, Boni Song, Sasha F. Malley, Calman A. MacLennan, Fan Zhang, Richard Malley, Ying-Jie Lu

**Affiliations:** 1 Division of Infectious Diseases, Boston Children’s Hospital, Harvard Medical School, Boston, MA 02115, USA; 2 Enteric & Diarrheal Diseases, Global Health, Bill & Melinda Gates Foundation, 500 5th Ave. N, Seattle, WA 98109, USA

**Keywords:** *Shigella*, MAPS vaccine, OSP, IpaB, quadrivalent

## Abstract

Background: Shigellosis is the leading cause of diarrheal deaths worldwide and is particularly dangerous in children under 5 years of age in low- and middle-income countries. Additionally, the rise in antibiotic resistance has highlighted the need for an effective *Shigella* vaccine. Previously, we have used the Multiple Antigen-Presenting System (MAPS) technology to generate monovalent and quadrivalent *Salmonella* MAPS vaccines that induce functional antibodies against *Salmonella* components. Methods: In this work, we detail the development of several monovalent vaccines using O-specific polysaccharides (OSPs) from four dominant serotypes, *S. flexneri* 2a, 3a, and 6, and *S. sonnei*. We tested several rhizavidin (rhavi) fusion proteins and selected a *Shigella*-specific protein IpaB. Quadrivalent MAPS were made with Rhavi-IpaB protein and tested in rabbits for immunogenicity. Results: Individual MAPS vaccines generated robust, functional antibody responses against both IpaB and the individual OSP component. Antibodies to IpaB were effective across *Shigella* serotypes. We also demonstrate that the OSP antibodies generated are specific to each homologous *Shigella* O type by performing ELISA and bactericidal assays. We combined the components of each MAPS vaccine to formulate a quadrivalent MAPS vaccine which elicited similar antibody and bactericidal responses compared to their monovalent counterparts. Finally, we show that the quadrivalent MAPS immune sera are functional against several clinical isolates of the serotypes used in the vaccine. Conclusions: This quadrivalent MAPS *Shigella* vaccine is immunogenicity and warrants further study.

## 1. Introduction

*Shigella* is the leading bacterial cause of diarrheal deaths worldwide and is associated with a large global burden of disease [1]. Shigellosis is particularly lethal in children under 5 years old in low- and middle-income countries (LMICs) with an estimated 93,000 child deaths out of 148,000 total deaths globally [2]. *Shigella* spreads by the fecal–oral route and contaminated food and water; only a small amount of bacteria, estimated to be between 10 and 100 colony-forming units, is necessary to cause disease [3]. A large number of *Shigella* serotypes can cause disease, therefore presenting a challenge for the development of serotype-specific vaccines [4]. *Shigella flexneri* is the most common diarrhea-causing species of *Shigella* in LMICs and consists of at least 15 serotypes. The most common disease-causing *S. flexneri* serotypes in LMICs are 2a, 3a, and 6. *Shigella sonnei* is dominant in developing countries and only has one serotype [5]. *Shigella dysenteriae* and *Shigella boydii* have been less frequently identified as causes of shigellosis in recent years. Antibiotic resistance is on the rise in *Shigella* isolates, further complicating therapeutic options [6]. For all these reasons, the development of an effective vaccine against Shigella, particularly one suited for deployment in LMICs, is an urgent priority [7].

Studies of natural *Shigella* infection have shown that protection after infection is serotype-specific, driven by O-specific polysaccharide antibodies [8,9]. A multivalent *Shigella* vaccine would be necessary to protect against a majority of diarrheal infections globally [1]. Data from the Global Enterics Multicenter Study (GEMS) suggest that quadrivalent vaccines containing O-antigens from *S. flexneri* 2a, 3a, and 6, and *S. sonnei* would offer excellent coverage while limiting the number of serotypes included [5,10]. The OSP repeating units for 2a and 3a share the same backbone of a tetrasaccharide (three rhamnose residues and one N-acetylglucosamine) and a branch α-D-Glcp from different rhamnose residues. The repeating units for 6 are two rhamnose residues, Galactopyranose and N-acetyl Galactopyranose. S. sonnei OSP is composed of two amino sugars: 2-acetamido-2-deoxy-l-altruronic acid (l-Alt*p*NAcA) and a 2-acetamido-4-amino-2,4,6-trideoxy-d-galactopyranose [11,12,13,14].

An alternative approach to O-antigen-based *Shigella* vaccines is the addition of Ipa proteins, such as IpaB, IpaC, and IpaD, which form part of the external portion of the *Shigella* type III secretion system that is essential for *Shigella* uptake into epithelial cells [15]. Immunization with IpaB can protect mice from invasive infection with *Shigella* strains [16]. The investigation of antibodies in human sera following natural exposure to and immunization with the auxotrophic live *Shigella flexneri* Y vaccine has demonstrated that Ipa proteins are immunogenic [17,18]. Controlled human infection model studies with candidate *Shigella* vaccines indicate that IgG to IpaB can correlate with protection [19,20]. 

In this work, we used the Multiple Antigen-Presenting System (MAPS) technology to develop a *Shigella* vaccine. The MAPS technology allows for the incorporation of pathogen-specific saccharides and proteins in a multivalent vaccine, through a biotin–rhizavidin interaction, as described previously [21]. We first evaluated and compared several fusion proteins for incorporation into the MAPS and selected the fusion protein IpaB. When IpaB fusion protein was affinity-linked to the OSP from the most prominent *Shigella* serotypes (*S. flexneri* 2a, *S. flexneri* 3a, *S. flexneri* 6, and *S. sonnei*) to create monovalent MAPS constructs, we showed that each generates robust IgG antibodies against the polysaccharide and protein in the vaccine. Using bactericidal assays, we demonstrated that immunization of rabbits with monovalent *Shigella* MAPS candidate vaccines generate functional antibodies against the relevant *Shigella* serotype. We also show that a combination of these four monovalent MAPS constructs into a quadrivalent MAPS vaccine elicits broad immune responses, including functional antibodies, against *Shigella*.

## 2. Materials and Methods

Bacterial strains and reagents: *Shigella flexneri* strains were obtained from Dr. Sharon Tennant from the University of Maryland. *Shigella sonnei* 53G was obtained from Dr. Eileen Barry from the University of Maryland. *Shigella sonnei* Mosely was obtained from Dr. Robert Kaminski from the Walter Reed Institute of Research in the US Army. 1-cyano-4-dimethylaminopyridinium tetrafluoroborate (CDAP) was acquired from Thermo Fisher (Waltham, MA, USA). Restriction endonucleases and T7 Shuffle Express competent cells were obtained from New England Biolabs (Ipswich, MA, USA). Aluminum phosphate (alum) was sourced from Brenntag North America (Reading, PA, USA). The plasmid pETDuet and additional reagents were purchased from Sigma (St. Louis, MO, USA).

DNA fragments encoding His-IpaB and IpgC (the chaperone for IpaB), or His-SseB and SseA (the chaperone for SseB), were codon-optimized, synthesized, and inserted into a pETDuet vector containing Rhizavidin (Rhavi) after digestion by restriction enzymes and ligation. Rhavi-IpaB or Rhavi-SseB was cloned between the NcoI and NotI sites, and IpgC or SseA was cloned between the SpeI and XhoI sites. The plasmids were sequencing-verified and introduced into *E. coli* T7 Shuffle Express cells. The transformed cells were grown to an OD600 of 1 at 30 °C, and the protein expression was then induced with 0.2 mM IPTG at 16 °C overnight. Cells were collected by centrifugation and pellets were resuspended in lysis buffer (20 mM Tris-HCl, 500 mM NaCl, pH 8.0). Cells were lysed by sonication. The supernatant was applied to a Ni-NTA column, and chaperone proteins were washed off with 0.1% sodium deoxycholate (SDOC) in lysis buffer. Rhavi-SseB and Rhavi-IpaB were eluted with lysis buffer containing 300 mM imidazole. The eluted proteins were pooled and further purified using a gel-filtration column. The dimer fractions were combined and used for MAPS assembly. 

OSP purification: OSPs were purified as prescribed previously with modifications [22]. Briefly, bacteria were grown in Luria Broth overnight at 37 °C 220 RPM and centrifuged at 5000 RPM for 10 min to collect cells. A 6% acetic acid solution was used to resuspend cell pellets. The resuspension was boiled for 3 h at 100 °C and then neutralized by adding ammonium hydroxide. After centrifuging at 12,000× *g* for 30 min, the supernatant was dialyzed extensively against water. Sample was concentrated by lyophilization and then treated with Q cassette and ethanol precipitation. Sample was dialyzed against water a final time and loaded on a gel-filtration column to separate the OSPs with larger sizes. The final OSP was lyophilized and frozen in −20 °C. The strain ID used for OSP purification were 200071 *S. flexneri* 2a, 603518 *S. flexneri* 3a, 603345 *S. flexneri* 6, *S. sonnei* 53G.

OSP quantification: The concentration of OSP was determined by the anthrone assay for *S. flexneri* 2a, 3a, and 6 [23]. Briefly, OSP samples were mixed with 1.5 mg/mL of anthrone in 98% sulfuric acid and incubated at 100 °C for 20 min. The reaction was cooled to room temperature and an OD reading was taken at 620 nm with an ELISA reader. The concentration of *S. sonnei* OSP was determined by HPAEC-PAD as described previously [24]. 

Biotinylation of OSP: *S. flexneri* 2a, *S. flexneri* 3a, *S. flexneri* 6, and *S. sonnei* OSP were biotinylated with CDAP using the protocol as described previously [21,25]. All procedures occur at room temperature. Amounts of 2 mg/mL OSP and 100 mg/mL CDAP were stirred for 30 s. An amount of 50 mM sodium borate was stirred into the reaction for 2 min. Amine-PEG3-biotin (40 mg/mL) was added at a ratio of 1:1 (*w*:*w*, OSP:biotin) and the reaction continued for 2 h. Glycine was added at a final concentration of 20 mM to stop the reaction. Biotinylated OSP was dialyzed against saline intensively. A No-weigh HABA/avidin Premix Biotin Quantification Kit was used to determine biotin concentration.

MAPS formulation: MAPSs were assembled by combining fusion proteins and biotinylated polysaccharides in a 4:1 (*w*:*w*) ratio, rotated overnight at 4 °C and run through a size-exclusion chromatography to separate MAPSs from free protein and OSP. MAPS fractions were combined, and protein and OSP concentrations were determined using the methods mentioned above. 

Antigen preparation and immunization procedures: MAPS vaccines were mixed with aluminum phosphate (alum, 0.5 mg/mL final concentration) and saline in a 5 mL tube, and then rotated end-over-end overnight at 4 °C. Rabbit immunization experiments were performed at Cocalico Biologicals Inc. (Denver, PA, USA). Rabbits were immunized intramuscularly with a dose equivalent to 5 μg of OSP per 500 μL injection two times with a three-week interval. Blood draw was taken before each immunization (Pre and P1) and three weeks after the last immunization (P2). All animal experiments were approved by the local Animals Care and Use Committee. 

Enzyme-linked immunosorbent assay (ELISA): IgG antibody titers against *S. flexneri* 2a, *S. flexneri* 3a, *S. flexneri* 6, and *S. sonnei* OSP and proteins IpaB, SseB, and CP1 were measured by ELISA as previously described [26,27]. Briefly, NUNC Maxisorp plates were coated overnight at room temperature. Coating antigens were 100 µL of proteins at 1 µg/mL or OSPS at 10 µg/mL in PBS. Plates were blocked with 200 µL of PBS 1% BSA for 1 h after washing with PBS/Tween solution (PBST). Serum samples were added and incubated for 2 h at room temperature. Plates were washed with PBST and then incubated with secondary antibody diluted 1:10,000 in PBST for 1 h. Plates were developed with Sureblue and stopped with 1M HCl. The absorbances were read at 450 nm.

Bactericidal assay (SBAs): The activity of antisera against *Shigella* strains in this study was analyzed with SBAs [28]. Briefly, dilutions of heat-inactivated rabbit serum were incubated with the relevant bacteria and complement for two hours at 37 °C. Surviving bacteria were enumerated after overnight incubation on blood agar plates. The killing titer is defined as the inverse of the lowest concentration of serum where 50% of the killing occurred. 

Opsonophagocytic assay (OPAs): Activity of antisera from IpaB-immunized rabbits was analyzed using OPAs [29,30]. Briefly, the serum was heat-inactivated before use. Serum was then diluted and incubated with target bacteria to allow the antibody to bind. Next, differentiated HL60 cells and baby rabbit complement were added to the reaction, and killing was continued at 37 °C for 2 h. The reaction was then terminated by the addition of Saponin to 1% final concentration. The surviving bacteria were counted after overnight incubation of the reaction on blood agar plates. The killing titer analysis was conducted the same as in the SBAs. 

Statistical analysis: Statistical analysis was carried out using the Mann–Whitney U test and PRISM (version 10.1, GraphPad Software, Inc., Boston, MA, USA).

## 3. Results 

### 3.1. Selection of Fusion Protein in S. flexneri 3a MAPS Complex

**Figure 1:**
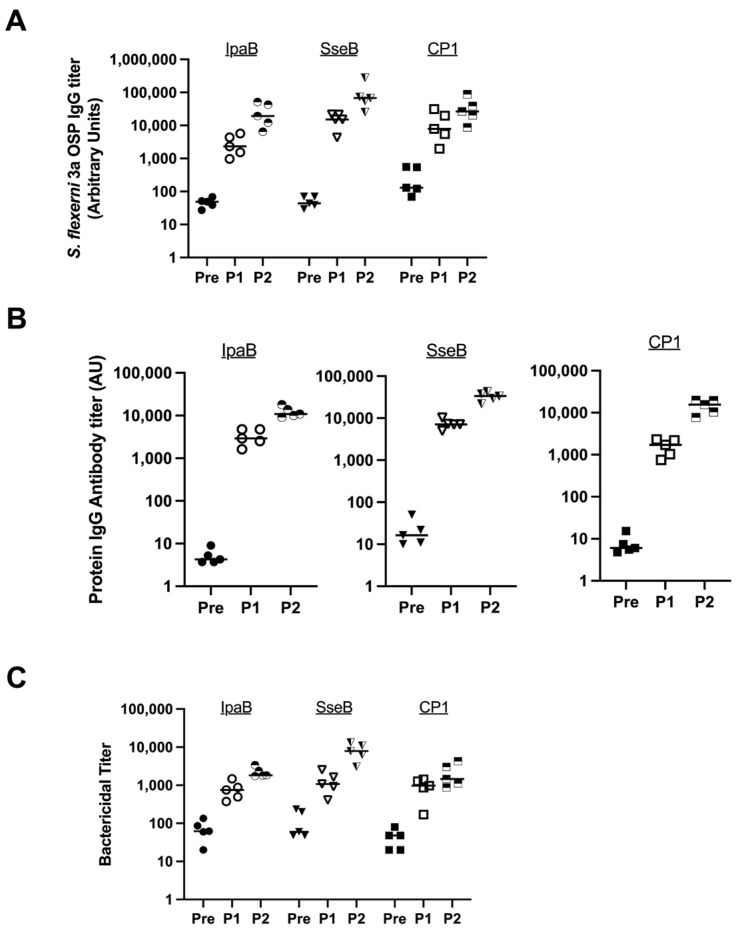
Comparison of different fusion proteins in MAPS formulations. Pre: rabbit sera prior to MAPS immunization (closed symbols). P1: rabbit sera post one immunization (open symbols). P2: rabbit sera post two immunizations (half-open symbols). (**A**) Measurement of *S. flexneri* 3a OSP IgG antibody titer generated from immunization of rabbits with different fusion proteins in the MAPS construct. (**B**) Protein IgG antibody titer analysis after MAPS constructs immunization. All three proteins are immunogenic. (**C**) Bactericidal assay to compare killing titers generated from sera of rabbits immunized with different MAPS constructs. Data were collected from two or more experiments; a representative result is shown. Isolate ID for SBA: 603518 *S. flexneri* 3a.

We randomly chose *S. flexneri* 3a to evaluate three fusion proteins to determine which was the most immunogenic MAPS complex with respect to the OSP. We evaluated Rhavi-IpaB, Rhavi-SseB (an analog of IpaB in *Salmonella* species), and CP1 (Rhavi-SP1500-SP0785, where SP1500 and SP0785 are two pneumococcal proteins; this fusion protein is included in a 24-valent pneumococcal MAPS vaccine currently in clinical trials) [30,31]. MAPS constructs were formulated at a 4:1 (*w*:*w*) ratio of protein to polysaccharide. Each immunization contained 5 µg polysaccharide/dose. In Figure 1, rabbits (n = 5 per group) were immunized with one of three MAPS vaccines twice three weeks apart; sera were collected before the first injection (Pre, solid symbols), three weeks after the first injection (P1, open symbols), and three weeks after the second injection (P2, half-filled symbols). IgG antibody titers against the OSP and relevant proteins in all sera were analyzed using ELISAs.

All three MAPS constructs generated antibodies against the *S. flexneri* 3a OSP, with responses representing more than a 100-fold increase from baseline after two doses (Figure 1A). There was no statistically significant difference between the antibody titers after two doses between the three MAPS constructs. All three fusion proteins were immunogenic (Figure 1B). The functionality of the antibodies was tested by serum bactericidal assays (SBAs). Each vaccine generated similar and robust killing titers against *S. flexneri* 3a (Figure 1C). 

**Figure 2:**
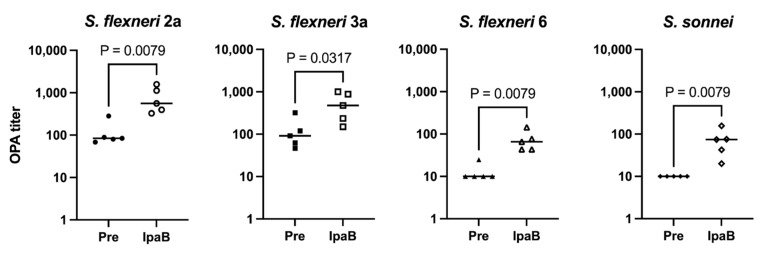
Ability of IpaB to generate functional antibodies. Pre: rabbit sera prior to IpaB immunization (closed symbols). IpaB: rabbit sera after two IpaB immunizations (open symbols). Opsonophagocytic activity of sera from rabbits immunized with IpaB against *S. flexneri* 2a, *S. flexneri* 3a, *S. flexneri* 6, and *S. sonnei*. Data were collected from two or more experiments; a representative result is shown. Isolate ID for OPA: 200071 *S. flexneri* 2a, 603518 *S. flexneri* 3a, 603345 *S. flexneri* 6, and *S. sonnei* 53G.

Due to the similar immunogenicity and functional antibody responses between each construct, we evaluated whether the inclusion of IpaB, a protein specific for *Shigella*, provides additional killing activity. To test this, opsonophagocytic assays (OPAs) were performed against all four *Shigella* serotypes. Figure 2 shows that immunization with IpaB generates some, albeit modest, killing activity against all four Shigella strains tested compared to sera obtained before immunization. We have also noticed some killing activity from pre-immunization sera against *S. flexneri* 2a and 3a strains, which leads to different background killing levels. Therefore, we chose IpaB as our fusion protein in the MAPS constructs, hoping that the induction of an anti-IpaB antibody would provide an additional protective effect in addition to the antibody against OSP, as shown from the analysis of human sera from controlled human infection model studies [20,32]. 

### 3.2. Immunogenicity and Functional Analysis of Antibodies after Immunization with Monovalent MAPS

**Figure 3:**
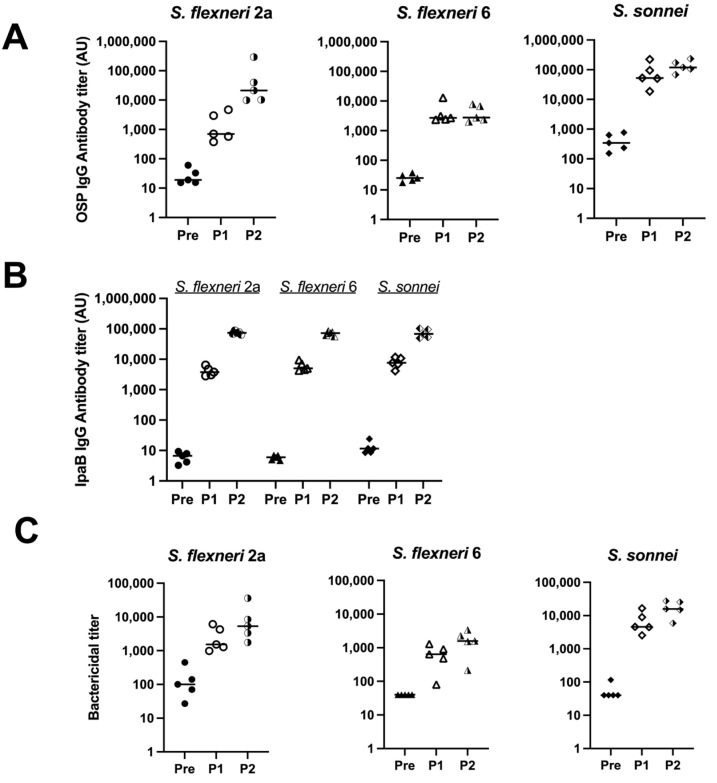
Quantity and function of IgG antibodies generated from rabbit immunization with monovalent MAPS. Pre: rabbit sera prior to MAPS immunization (closed symbols). P1: rabbit sera post one immunization (open symbols). P2: rabbit sera post two immunizations (half-open symbols). (**A**) *S. flexneri* 2a, *S. flexneri* 6, and *S. sonnei* OSP antibody titers after each monovalent MAPS constructs rabbit immunization. (**B**) IpaB IgG antibody titer analysis after each monovalent MAPS constructs immunization. (**C**) Bactericidal assays to determine activity of antibodies in rabbit sera after one and two doses of MAPS constructs. Data were collected from two or more experiments; a representative result is shown. Isolate ID for SBA: 200071 *S. flexneri* 2a, 603345 *S. flexneri* 6, *S. sonnei* 53G.

To test the immunogenicity of the MAPS with respect to *S. flexneri* 2a, *S. flexneri* 6, and *S. sonnei*, OSPs were purified from each organism. MAPS constructs were formed using IpaB as the rhizavidin fusion protein. The serum antibody levels against each OSP were analyzed after each rabbit immunization. Robust IgG antibody titers were generated after two doses of each vaccine (Figure 3A, half-filled symbols). The antibodies are specific to each organism within the MAPS, with little to no cross-reactivity between species and serotypes (Figure S1). Each monovalent MAPS construct elicited robust antibodies to the IpaB protein. (Figure 3B). The functionality of generated antibodies was analyzed with SBAs with each organism (Figure 3C). Each MAPS construct individually generated functional antibodies against their respective organism. These data, taken together with Figure 1, demonstrate that MAPS constructs including OSP from *S. flexneri* 2a, *S. flexneri* 3a, *S. flexneri* 6, or *S. sonnei* and IpaB elicit significant levels of functional antibodies to each component. 

### 3.3. Quadrivalent Shigella MAPS Vaccine

**Figure 4:**
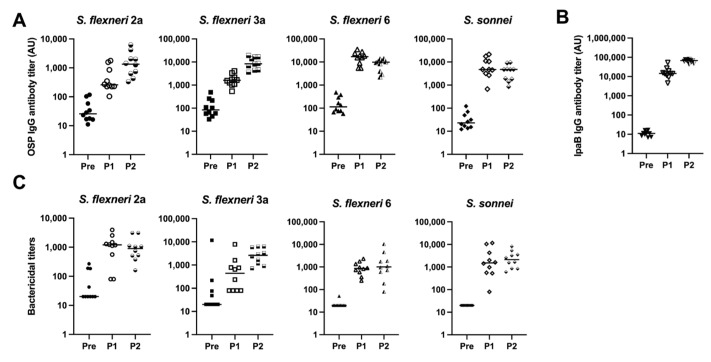
Analysis of rabbit sera after immunization with Quadrivalent *Shigella* MAPS. Pre: rabbit sera prior to MAPS immunization (closed symbols). P1: rabbit sera post one immunization (open symbols). P2: rabbit sera post two immunizations (half-open symbols). (**A**) *S. flexneri* 2a, *S. flexneri* 3a, *S. flexneri* 6, and *S. sonnei* OSP antibody titers after two rabbit immunizations of quadrivalent MAPS vaccine. (**B**) IpaB IgG antibody titer analysis after two quadrivalent MAPS vaccine immunizations. (**C**) Bactericidal assays to determine activity of antibodies in rabbit sera after two doses of quadrivalent MAPS construct. Data were collected from two or more experiments; a representative result is shown. Isolate ID for SBA: 200071 *S. flexneri* 2a, 603518 *S. flexneri* 3a, 603345 *S. flexneri* 6, *S. sonnei* 53G.

**Figure 5:**
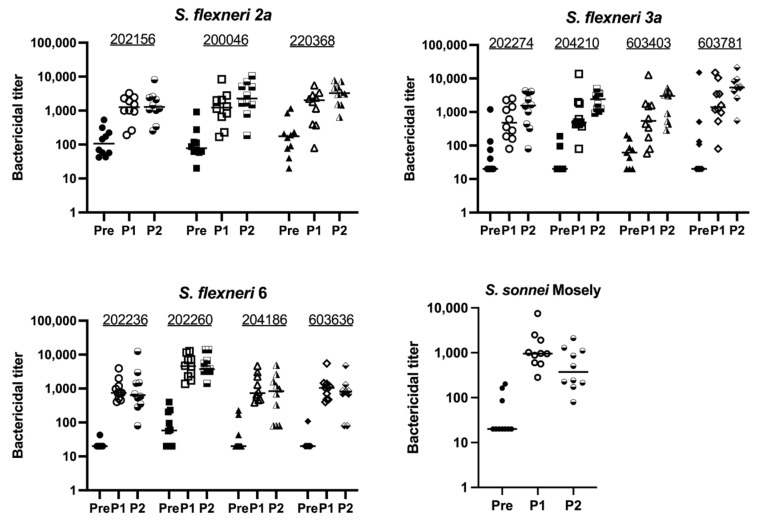
Bactericidal activity of Quadrivalent *Shigella* MAPS immunized rabbit sera against different clinical strains. Pre: rabbit sera prior to MAPS immunization (closed symbols). P1: rabbit sera post one immunization (open symbols). P2: rabbit sera post two immunizations (half-open symbols). Sera from rabbits immunized with quadrivalent *Shigella* MAPS show similar bactericidal activity against other clinical strains of the same serotypes. Data were collected from two or more experiments; a representative result is shown. Isolate IDs are listed on graphs.

Given the immunogenicity of the monovalent MAPS vaccines, we combined the four monovalent MAPS constructs into one vaccine and immunized rabbits with 5 µg per OSP per dose. The OSP antibody titers were similar to their monovalent counterparts after two doses (Figure 4A). IpaB was immunogenic as expected (Figure 4B). The function of OSP IgG antibodies generated with the quadrivalent MAPS vaccine was studied using bactericidal assays and demonstrated similar killing activity to the monovalent MAPS (Figure 4C). The quadrivalent MAPS sera also provided robust killing against additional clinical strains of each serotype (Figure 5). These data suggest that a quadrivalent *Shigella* MAPS vaccine is immunogenic and capable of generating functional antibodies to target several clinical strains of the homologous serotype. 

## 4. Discussion

A vaccine against prominent *Shigella* serotypes could aid in controlling the burden of shigellosis in LMICs. A *S. sonnei* O-antigen glycoconjugate vaccine was developed by the National Institutes for Health (NIH) and protected Israeli military recruits and children older than three years [33,34]. This work laid the foundations for the development of second-generation O-antigen-based parenteral *Shigella* vaccines [7]. There are several vaccines being evaluated clinically and preclinically, targeting different *Shigella* serotypes. 

Conjugate and subunit vaccines are being actively tested, due to their low reactivity and in theory a robust immunogenic response [7]. The bioconjugate *S. flexneri* 2a candidate vaccine, Flexyn2a, has been shown to be safe, immunogenic, and protective against moderate to severe shigellosis in a controlled human intentional challenge model (CHIM) study (NCT02646371, NCT02388009) [35,36]. This vaccine technology is now being used in a tetravalent bioconjugate candidate vaccine Shigella4V by LimmaTech Biologics AG to study its safety, immunogenicity, and dose in Kenyan infants (NCT04056117). Invaplex_AR_ and Invaplex_AR-DETOX_ are subunit vaccines containing IpaB, IpaC, and either wild-type LPS or under-acylated LPS, respectively [37]. Both were safe and immunogenic in phase I studies (NCT02445963, NCT03869333). Invaplex_AR_ generated bactericidal antibodies against *S. flexneri* 2a and other *Shigella* species and antibodies were present in the serum of test subjects almost 18 months later [37,38]. Further clinical studies are being designed to evaluate immunogenicity in target populations. Finally, preclinical studies and a Phase 1 study of synthetic carbohydrate-based conjugate vaccine called Sf2a-TT15, a chemically synthesized oligosaccharide linked to tetanus toxoid, yielded promising results [39]. Currently, Sf2a-TT15 is being evaluated in young children and infants in Kenya (NCT04602975), and a 10ug O-antigen equivalent dose vaccine is being evaluated in a CHIM study in North American adults (NCT04078022).

The GSK Vaccines Institute for Global Health (GVGH) has been working on two Generalized Modules for Membrane Antigens (GMMA) vaccines to target *Shigella*. A *Shigella sonnei* GMMA vaccine, 1790GAHB, was well tolerated in two Phase 1 studies in healthy adults (NCT02017899, NCT02034500) [40], but gave disappointing results in a CHIM study (NCT03527173) [41]. A Phase 1/2 clinical study of the 4-valent GVGH altSonflex 1-2-3 GMMA vaccine is underway to determine the appropriate dose for further vaccine development (NCT05073003) [4]. 

Based on the experience with the pneumococcal MAPS, the MAPS technology elicits a strong immune response to each of included antigens, is well tolerated, and is economic to produce [31,42]. Using the MAPS technology, we combined two immunogenic *Shigella* antigens, IpaB and O-specific polysaccharides, from four dominant serotypes. 

Carrier proteins are important for the induction of anti-polysaccharide antibodies for conjugate vaccines [43]. Thus, we have tested different fusion proteins for the shigella MAPS, as we have done previously for the bivalent Vi/Paratyphi A vaccine and the quadrivalent Salmonella Vaccine [29,30]. Consistently with our previous results, the MAPS made with all three fusion proteins induced good anti-OSP antibody which are bactericidal (Figure 1). The inclusion of IpaB appears to generate some cross-serotype opsonophagocytic killing activity (Figure 2), but clinical studies would be required to confirm this effect in humans. We then tested Rhavi-IpaB in combination with the other three OSPs from *S. flexneri* 2a, 6, and *S. sonnei*, and found that MAPS immunization generated a high level of antibodies against the three OSPs and the antibodies were highly functional (Figure 3). We also showed that the antibodies generated against these OSPs are very specific, and not much cross-activity was observed (Figure S1).

Combining the four monovalent MAPSs into one quadrivalent vaccine yielded functional antibodies similar to those generated by monovalent constructs (Figure 4). Thus, this suggests that there is little or no interference between these MAPSs. Our quadrivalent *Shigella* MAPS generates antibodies that exhibit successful bactericidal activity against several clinical strains of the same serotypes (Figure 5), which further proves the specificity of MAPS sera against the included serotypes. In addition, the killing activity of MAPS sera against clinical strains suggests that MAPS vaccine may be functional in clinical settings. While these antibodies demonstrate bactericidal activity in vitro, it is unknown to what extent these will correlate with protection from disease in humans and particularly in young children. 

## Data Availability

Data are available upon request.

## Supplementary Materials

The following supporting information can be downloaded at: https://www.mdpi.com/article/10.3390/vaccines12101091/s1, Figure S1: Analysis of OSP IgG antibody specificity to parent strain’s OSP included in MAPS formulation.
