# Supplementary material for: Evaluation of a Quadrivalent Shigella flexneri Serotype 2a, 3a, 6, and Shigella sonnei O-Specific Polysaccharide and IpaB MAPS Vaccine
Source: Vaccines (Basel). 2024 Sep 24;12(10):1091. doi: 10.3390/vaccines12101091 (PMC11510904; doi:10.3390/vaccines12101091)
Supplement: Supplementary file 1 [file vaccines-12-01091-s001.zip › vaccines-3161958-supplementary.pdf]

Supplementary Material

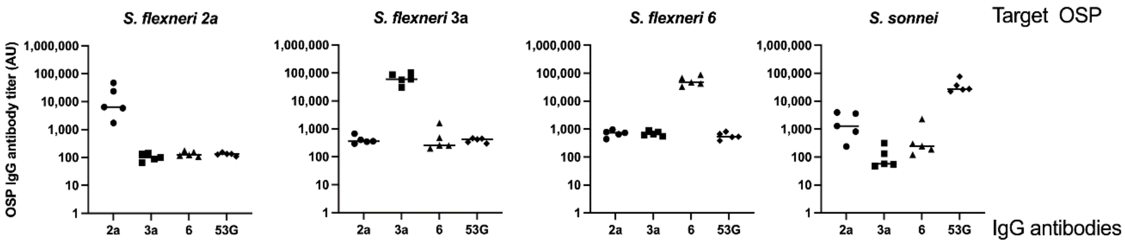

Figure S1. Analysis of OSP IgG antibody specificity to parent strain's OSP included in MAPS formulation. IgG antibodies from the four monovalent MAPS were tested against each of the parent OSPs to determine the specificity of the antibodies to each OSP. The three *Shigella flexneri* OSPs were only recognized by antibodies generated from matching MAPS immunizations. *Shigella sonnei* OSP was recognized by *S. sonnei* MAPS antibodies and some cross recognition by *S. flexneri* 2a antibodies. Data were collected from two or more experiments; a representative result is shown.
